# Drug utilization and cost in a Medicaid population: A simulation study of community vs. mail order pharmacy

**DOI:** 10.1186/1472-6963-7-122

**Published:** 2007-07-30

**Authors:** Satish Valluri, Enrique Seoane-Vazquez, Rosa Rodriguez-Monguio, Sheryl L Szeinbach

**Affiliations:** 1 College of Pharmacy, The Ohio State University, 500 West 12th Avenue, Columbus, OH 43210, USA; 2 Center for Health Outcomes, Policy and Evaluation Studies, College of Public Health, The Ohio State University, 1841 Millikin Road, Columbus, OH 43210, USA; 3 School of Public Health and Health Sciences, University of Massachusetts, 715 North Pleasant St, Amherst, MA 01003, USA

## Abstract

**Background:**

Outpatient drugs are dispensed through both community and mail order pharmacies. There is no empirical evidence that substitution of community pharmacy with mail order reduces overall drug expenditures. The need for evaluating the potential effects on utilization and costs of the possible extension of mail order services in Medicaid provides the rationale for conducting this study. This study compares drug utilization and drug product cost in community vs. mail order pharmacy dispensing services in a Medicaid population.

**Methods:**

This study is a retrospective cohort study comparing utilization and cost patterns in community vs. mail order pharmacy. A simulation model was employed to assess drug utilization and cost in mail order pharmacy using community pharmacy claim data. The model assumed that courses of drug therapy (CDT) in mail order pharmacy would have utilization patterns similar to those found in community pharmacy. A 95% confidence interval surrounding changes in average utilization and average cost were estimated using bootstrap analysis. A sensitivity analysis was performed by varying drug selection criteria and supply, fill point, and medication possession ratio (MPR). Sub-analyses were performed to address differences between mail order and community pharmacy related to therapeutic class and dual-eligible patients.

Data for the study derived from pharmacy claims database of Ohio Medicaid State program for the period January 2000-September 2004. Drug claims were aggregated to obtain a set of CDTs representing unique patient IDs and unique drug products. Drug product cost estimates excluded dispensing fees and were used to estimate the cost reduction required in mail order to become cost neutral in comparison with community pharmacy.

**Results:**

The baseline model revealed that the use of mail order vs. community pharmacy would result in a 5.5% increase in drug utilization and a 5.4% cost reduction required in mail order to become cost neutral. Results from Ohio Medicaid drugs for chronic use revealed a 5.1% increase in utilization and a 4.9% cost reduction required to become cost neutral in comparison with community pharmacy.

**Conclusion:**

The results of the simulation model indicate that mail order pharmacy increases drug utilization and can also increase drug product cost if the cost per unit is not reduced accordingly. Prior consideration should be given to the patient population, day-supply, disease, therapy, and insurance characteristics to ensure the appropriate use of mail order pharmacy services.

## Background

Outpatient drugs are dispensed through both community and mail order pharmacies. In 2004, mail order pharmacies dispensed 214 million prescriptions, representing 6.5% of the prescriptions dispensed in the U.S. outpatient pharmaceutical sector during that year [1]. Prescriptions for mail order provide a 90-day supply as opposed to a 30-day supply typically dispensed in community pharmacies. When mail order prescriptions are converted to 30-day supplies, mail order pharmacies can be viewed as having dispensed an estimated 642 million prescriptions in 2004, representing 17.3% (up from 12% in 1994) of the outpatient market. These numbers translate into $41.3 billion in sales, representing 18.7% of U.S. outpatient prescription sales [1].

Initially, mail order pharmacy was perceived as an alternative for patients who use a high volume of prescription drugs for chronic conditions [2-6] or for patients who cannot access community pharmacy services [7,8]. Although cost containment is the main reason behind the recent development of mail order pharmacy [3,9,10], there is no empirical evidence that substitution of community pharmacy with mail order reduces overall drug expenditures.

Increases in utilization could result in higher overall prescription drug expenditures for mail order pharmacy in spite of a lower cost per unit. Previous studies found that drug utilization in pharmacy benefit managers (PBM) commercial populations was higher in mail order pharmacy than in community pharmacy[3,9,10]. Higher drug utilization in mail order pharmacies is attributed to an established drug supply of 90 days and because the co-payments associated with mail order are lower than those associated with community pharmacy [11]. However, no studies have been done to control for patient characteristics that could influence utilization. For example, patients that have better adherance to drug therapy could be more inclined to select mail order pharmacy.

In addition, drug therapy discontinuation is frequent regardless of the therapy utilized [12-18], and such discontinuation can lead to wastage in cases where patients do not complete their drug regimens. Both increased utilization and increased wastage (regardless of the reasons for discontinuation) are expected in mail order pharmacy. Moreover, as an incentive to use mail order pharmacy services, higher co-payments, third-party rejections for drug refills, and day-supply controls may be implemented if a plan enrollee obtains the drug from a community pharmacy.

The scope of this study is a Medicaid population. Mail order services have been implemented in the Medicaid programs of Maine and Washington [19], and multiple states considered mail order as an alternative pharmacy delivery system for their Medicaid programs. The need for evaluating the potential effects on utilization and costs of the possible extension of mail order services in Medicaid provides the rationale for conducting this study. Additionally, Medicaid/Medicare dual eligible patients included in the Medicare Part D program after January 2006 are managed by private plans, which use mail order services more often.

Mail order services in Medicaid have not been examined previously in the scientific literature. Medicaid differs from traditional comercial plans in that benefits are controlled at both the federal and state levels. Moreover, Medicaid programs are managed by the state departments with PBM support for claims processing and other services. Also, state Medicaid programs define their own pharmacy reimbursement structures, including the estimated drug acquisition cost and dispensing fees. Finally, Medicaid drug pricing is specially influenced by pharmaceutical company rebates, which are regulated at the federal level, and most states have a supplemental rebate program.

The objectives of this study are: 1) to compare drug utilization in a Medicaid population using community pharmacy services with the results of a model simulating the effect of mail order pharmacy in the same population; and 2) to estimate the mail cost reduction required to become cost neutral in comparison with community pharmacy.

## Methods

This study is a retrospective cohort study comparing utilization and cost patterns in community and mail order pharmacy. Drug utilization is defined in this study as the day supply of drug product reimbursed by the Ohio Medicaid program A novel simulation model using community pharmacy claim data was employed to assess drug utilization in mail order pharmacy and mail order cost reduction required to become cost neutral in comparison with community pharmacy. The model used the course of drug therapy (CDT) representing unique patient IDs and unique drug products (i.e. generic name(s), formulation, and strength(s)) as the unit of analysis. The cost was estimated from the perspective of the Medicaid program.

Pharmacy claims data from the Ohio Medicaid program for the period January 2000-September 2004, including 93.0 million unduplicated claims for patients who were not institutionalized at any point during the study period were used for the analysis (Table 1). The data set was aggregated to obtain a final set of CDTs representing unique patient IDs and unique drug products (i.e. generic name(s), formulation, and strength(s)). The final data set contained 18.5 million CDTs, with an average of five claims per CDT. The Ohio State University's Institutional Review Board granted exemption for the study.

**Table 1:** Ohio Health Plans- Pharmacy Claim Data

	Number	% of Total
Institutionalized patient claims	39,028,913	29.55%
Community pharmacy claims	93,029,178	70.45%
Unduplicated claims	132,058,091	100.00%

Drug therapies (community pharmacy)	18,544,752	

Average claims per drug therapy	5.02	

All CDTs initiated between August 2000 and July 2001 were selected for inclusion in the study. CDTs initiated between January and July 2000 were excluded to ensure that the drug therapy was newly provided (i.e. that patients had not taken the therapy during the six months prior to selection). Therapies initiated after July 2001 were excluded to allow for a follow-up period of at least three years and two months and to minimize right censoring data issues. The baseline analysis excluded chronic drugs for short-term use and included acute drugs for chronic use.

The criteria for selecting the baseline sample from the final data set was that, for each CDT, patients should have utilized a supply of at least 90 days and should have maintained a Medication Possession Ratio (MPR) of at least 0.5. The MPR was estimated using the number of days supply reimbursed by Ohio Medicaid according with the information available in the claims data. Although the MPR does not assess actual consumption, it is a proxy measure of adherence that determines the proportion of days a patient has medication available to be taken [20]. The MPR for each CDT was calculated by dividing the day supply for all fills except the last fill by the total number of days between the first and last fill. MPR values above 1.0 indicate that the patient is obtaining early refills, taking more medication than that provided in the defined daily dosage, or the day supply was processed incorrectly at the pharmacy.

The mail order simulations did not include the first fill, because mail order pharmacy starts typically with the first refill. The effect of mail order on drug utilization was calculated for each CDT by dividing the MPR-adjusted community pharmacy day supply by 90 and rounding that result to the next highest integer. Incremental changes in drug utilization attributable to the effect of mail order were estimated.

The methodology utilized in the mail order simulation model can be illustrated by the following example (Table 2): In the case of a patient who has received 600 day supply after the first fill in community pharmacy during the study period, who has also maintained a MPR of 1 for the entire period, the 600 day supply is divided by 90 (given that mail order pharmacy provides a 90 day supply for each fill); the quotient is rounded to 7 (the next highest integer); and the product of 90 and 7 is multiplied by the MPR (i.e. 1), resulting in 630. The incremental change in drug utilization attributable to the effect of mail order is the difference between the 630 mail order day supply and the 600-community-pharmacy day supply, that is, a positive 30 day supply representing a 5% increase in utilization. Using the same example, a change in the MPR would result in a different mail order utilization amount.

**Table 2:** Effect of Medication Possession Ratio (MPR) on Mail Order Modeling Results

	**Example 1. MPR Equal to 1**			**Example 2. MPR Equal to 1.25**			**Example 3. MPR Equal to .75**		
	
	CP	MO	Diff.	CP	MO	Diff.	CP	MO	Diff.
Total day supply	600	630	30	750	788	38	450	450	0
MPR	1.00	1.00	0.00	1.25	1.25	0.00	0.75	0.75	0.00
Days	600	630	30	600	630	30	600	600	0
Refills	20	7	-13	20	7	-13	15	5	-10
30-day-equivalent refills	20	21	1	20	21	1	15	15	0
Average day supply per refill	30	90	60	38	113	75	30	90	60

CP = Community Pharmacy; MO = Mail order pharmacy; Diff. = Difference

Note: Numbers are rounded to the next integer.

The mail order pharmacy cost reduction required to become cost neutral in comparison with community pharmacy were estimated by first calculating the drug product cost per claim in community pharmacy; that is, by deducting the Ohio State Medicaid dispensing fee of $3.70 per claim. Next, community pharmacy drug product cost per day of supply was calculated for each CDT by dividing the drug product cost by the total number of days the drug was supplied. It was assumed that drug product cost in community and mail order pharmacy would be the same. This assumption allowed for the estimation of cost differences between both channels and the assessment of the cost reduction that mail order should achieve to become cost neutral. Hence, drug product cost in mail order pharmacy was estimated by multiplying the mail order day supply figure by the community pharmacy drug product cost per day supply. Costs were not adjusted for inflation or discounted. As with the estimates of drug utilization, estimates of differences in drug product cost did not take into account the day supply associated with patients' first fill.

Once the incremental changes in utilization attributable to the use of mail order were modeled and the mail order cost reduction required to become cost neutral in comparison with community pharmacy, a 95% confidence interval surrounding these changes was constructed. Specifically, bootstrap analysis, a non-parametric approach used to avoid distributional assumptions [21] was used to estimate the 95% confidence interval surrounding estimated changes in average utilization and average cost in order to address the lack of normality in the distribution of the utilization and cost data.

Sub-analyses were performed in relation to drugs for chronic conditions (as determined by State Medicaid drug classifications), dual-eligible patients (Medicare/Medicaid patients), and that subset of dual-eligible patients using drugs for chronic conditions. The sub-analyses utilized a baseline sample based on the selection criteria described above. Similar to the main analysis, sub-analyses also calculated incremental changes in utilization attributable to mail order and the mail order cost reduction required to become cost neutral in comparison with community pharmacy.

Multivariate sensitivity analysis was performed in relation to the baseline model by varying the following parameters: 1) point where mail order filling was initiated (i.e., whether first fill, second fill, etc.), 2) minimum acceptable MPR for a CDT being eligible for mail order, and 3) minimum day supply utilized in community pharmacy for a CDT being eligible for mail order.

## Results

The baseline model included 258,412 patients, 680,277 CDTs, and 869 drugs in 26 therapeutic classes. Demographic characteristics were estimated at the patient and CDT levels (Table 3).

**Table 3:** Baseline Sample Characteristics

Variables	Patients	% of Patients	CDT*	% CDT
Observations	258,412		680,277	

Gender				
Female	169,219	65.50%	460,975	67.80%
Male	89,193	34.50%	219,302	32.20%

Average Age (Yrs.)	43.1(± 22.0)		49.4(± 20.0)	

Ethnicity				
White	200,231	77.50%	532,054	78.20%
Black	53,922	20.90%	137,949	20.30%
Asian	1,641	0.60%	3,859	0.60%
Other	2,618	1.00%	6,415	0.90%

Hispanic (of any race)	4,146	1.60%	9,683	1.40%

Program				
Aged, Blind, and Disabled	104,348	40.40%	323,780	47.60%
Dual-Eligible	60,591	23.40%	199,902	29.40%
Healthy Start	64,703	25.00%	98,347	14.50%
Other	28,770	11.10%	58,248	8.60%

Note: CDT – Courses of Drug Therapy

**Figure 1:**
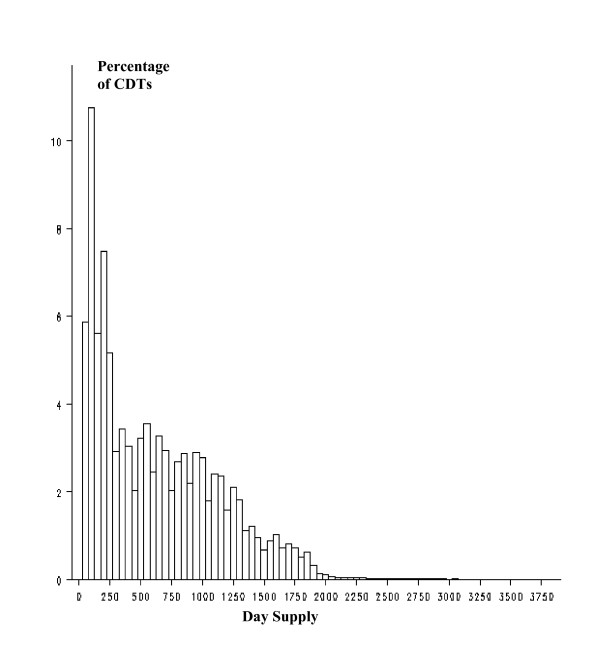
Distribution of day supply per CDT. Community pharmacy.

**Figure 2:**
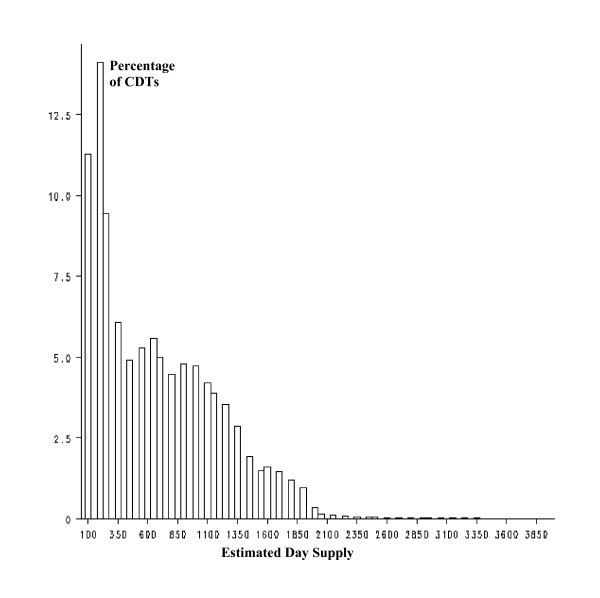
Simulated distribution of day supply per CDT. Mail order.

The distribution of number of day supply per CDT was right-skewed for both community and mail order pharmacy (Figures 1 and 2), reflecting a higher ratio of discontinuation of drug therapies during the first year of therapy.

The final sample of 680,277 CDTs yielded a mean of 640.0 ± 515.0 day supply in community pharmacy and the model for mail order resulted in 675.0 ± 518.0 day supply (Table 4). The mean difference was estimated at 35.3 days supply (95% confidence interval of the mean difference = 32.5–35.3).

**Table 4:** Changes in Utilization and Cost Attributable to Mail Order. Baseline Model (n = 680,277)

	Mean	Std Dev	Confidence Interval of the Mean Difference (95%)	
			Lower	Upper
Day supply in community pharmacy	640.0	515.0		
Estimated day supply in mail order pharmacy	675.0	518.0		
Difference	35.3		35.2	35.3
% Difference	5.52%			

Drug product cost in community pharmacy	$1,395.63	$5,982.19		
Estimated drug product cost in mail order pharmacy	$1,471.62	$6,349.17		
Mail order cost reduction required to become cost neutral	$75.99		$75.97	$77.00
% Difference	5.44%			

Note: Utilization and costs after the first fill.

The mean cost in community pharmacy of the CDTs included in the sample was $1,395.63 ± $5,982.19 and the model for mail order resulted in a mean cost of $1,471.62 ± $6,349.17. The mean mail order cost reduction required to become cost neutral in comparison with community pharmacy was estimated at $75.99 (95% confidence interval of the mean difference = $75.97–$77.00).

Thus, a 5.5% increase in drug utilization was associated with the use of mail order. This increase in utilization would lead to an estimated 5.4% mail order cost reduction required to become cost neutral in comparison with community pharmacy over the follow-up period that started in January 2000 and ended in July 2004 (Table 4).

When the sub-analysis on drugs classified by the Ohio State Medicaid Program as drugs for chronic use was performed, mail order resulted in an average day supply increase of 34.8 days, representing a 5.1% increase in utilization and a 4.9% cost reduction required to become cost neutral (Table 5). The use of mail order by dual-eligible patients was estimated to result in a day supply increase of 35.4 days, representing a 4.7% increase in utilization and a 4.7% cost reduction required to become cost neutral Finally, the use of mail order by dual-eligible consuming drugs for chronic use was estimated to result in a day supply increase of 34.8 days, representing a 4.3% increase in utilization and a 4.4% cost reduction required to become cost neutral. The difference between the baseline analysis and the sub-analyses is due to the increase in average day supply per CDT.

**Table 5:** Changes in Utilization and Cost Attributable to Mail Order. Drugs for Chronic Use and Dual-Eligible

	Drugs for Chronic Use (n = 304,102)		Dual-Eligible (n = 199,902)		Dual-Eligible Using Drugs for Chronic Use (n = 96,868)	
	Mean	Std Dev	Mean	Std Dev	Mean	Std Dev

Day supply in community pharmacy	687.6	523.2	758.8	527.8	807.0	526.7
Estimated day supply in mail order pharmacy	722.4	525.2	794.2	530.1	841.8	528.7
Difference	34.8		35.4		34.8	
% Difference	5.1%		4.7%		4.3%	

Drug product cost in community pharmacy	$1,103.65	$2,918.83	$1,563.01	$7,114.38	$1,219.59	$2,139.52
Estimated drug product cost in mail order pharmacy	$1,157.30	$3,039.04	$1,635.63	$7,531.51	$1,272.62	$2,226.44
Mail order cost reduction required to become cost neutral	$53.65		$72.62		$53.03	
% Difference	4.9%		4.7%		4.4%	

Note: Utilization and costs after the first fill

The model was sensitive to assumptions related to the minimum community pharmacy day supply required for mail order eligibility (Table 6). Keeping all other baseline variables constant, drug utilization in mail order increased 7.1% when the minimum day supply required for mail order eligibility was assumed to be 30. The incremental increase in utilization fell below 5% when the minimum day supply required for mail order eligibility was increased to 180 days or when the minimum MPR was increased to 0.75.

**Table 6:** Sensitivity Analysis

Scenario	Minimum MPR	Fill at Which Mail Order Is Initiated	Minimum Community Pharmacy Day Supply Required for Mail Order Eligibility	Incremental Change in Drug Utilization (%)	Mail order cost reduction required to become cost neutral (%)
Baseline	0.50	2	90	5.5%	5.4%
1	0.33	2	90	6.1%	6.0%
2	0.50	1	90	5.4%	5.3%
3	0.50	2	30	7.1%	7.1%
4	0.50	2	60	6.4%	6.3%
5	0.50	2	120	5.2%	5.2%
6	0.50	2	150	5.1%	5.0%
7	0.50	2	180	4.8%	4.7%
8	0.50	3	90	5.9%	5.9%
9	0.75	2	90	4.2%	4.2%

## Discussion

In this study a model was developed to assess utilization and drug costs to compare community and mail order pharmacy services using the same group of patients. Differences in drug utilization for mail order and community pharmacy as shown in the model are consistent with findings from previous studies that found an increase in utilization associated with the use of mail order pharmacy[3,9,10] These findings were gleaned from the comparison of two groups of patients from the same plan, one group using mail and other group using community pharmacy services. The simulation model in this study was developed using a case-crossover design within the specific parameters of a Medicaid population, thus minimizing the chance of selection bias.

The results of the sub-analyses indicate that the increase in drug utilization in mail order remained relatively constant (at a day supply of approximately 35 days) regardless of the sample utilized (i.e., all CDTs, CDTs utilizing drugs for chronic conditions, or CDTs observed in dual-eligible patients). Nevertheless, an increase in total day supply per CDT was observed in the sub-analyses, which resulted in a lower relative difference in utilization in mail order vs. community pharmacy. This reduction in the utilization relative difference has implications for mail order's impact on cost: The lower the overall relative difference in utilization, the lower the overall difference in unit cost must be for mail order pharmacy to become cost-neutral.

The results of the sensitivity analysis indicate that a reduction in the minimum number of day supply the CDTs were utilized in community pharmacy to become eligible for mail order increased the difference in utilization between mail order and community pharmacy. This result was the expected because the reduction in the number of day supply allowed for inclusion in the model of medications used for short term purposes. Finally, increases in the MPR reduced utilization relative differences, indicating that mail order is more appropriate for patients with high compliance.

Information about the unit cost of mail order in Medicaid is not available therefore, we assumed that the cost would be the same in mail and in community pharmacy regarding the estimation of the breakeven point; that is the cost reduction required in mail order to become cost neutral in comparison with community pharmacy. The results of our study indicate that mail order cost should be reduced in 5.4% to breakeven with the cost in community pharmacy.

Mail order pharmacy has cost advantages and disadventages over community pharmacy. First, dispensing a 90 day supply prescription instead of three 30 day supply prescriptions reduces dispensing costs for mail order operations. However, this reduction in dispensing costs could also be expected in community pharmacy if a 90 day supply were dispensed instead of a 30 day supply. Second, large mail order operations may have lower drug product acquisition cost than the average community pharmacy. With commercial plans, the cost per unit is lower in mail order than in community pharmacy due to a reduction in drug product cost, lower dispensing fees and higher rebates. However, mail order pharmacy could experience higher overall cost per CDT for the Medicaid program if drug product costs, supplemental rebates and/or dispensing fees are not adjusted accordingly with the increase in utilization. In addition, wholesaler distribution and inventory costs may also be lower in mail order pharmacy than in community pharmacy. However, mail order pharmacy has costs that are not found in community pharmacy, including packaging, mailing, and special handling costs, and the cost of drugs lost in mail.

Third, plan benefits may be structured according to pharmacy characteristics and geographical area. For example, pharmacy reimbursement in the Medicaid programs of Maine and Washington is lower for mail order than for community pharmacy (Table 7). An examination of the effect of mail order on pharmacy expenditures for these two programs would require the analysis of the estimated acquisition costs, professional and dispensing fees, co-payments, and administrative costs paid by the programs. Additionally, the study does not account for drug cost management tools that are implemented to some extent differently at mail and community pharmacies.

**Table 7:** Medicaid Pharmaceutical Reimbursement Structure in Maine and Washington, Quarter Ending March 2006

	**Maine**	**Washington**
**Drug Product Cost**	**Community** General: AWP-15% Direct supply drug list:* Usual & customary charge or AWP-17% plus $3.35 professional fee or FUL or MAC plus $3.35 professional fee **Mail order** Lowest of: usual & customary charge, AWP-20% plus $1.00 professional fee, or FUL or MAC plus $1.00 professional fee	**Community** AWP-14% single source & multiple source (w/2–4 manufacturers) AWP-50% multiple source from 5+ manufactures **Mail order** AWP-19% (brand-mail order), AWP-15% (generic-mail order)

**Dispensing Fee**	**Community and Mail Order** $3.35; $4.35 5.35(compounding);$12.50 (insulin syringe)	**Community** $4.20–$5.20 **Mail order** $3.25

**Co-Payment**	**Community** $2.50 generic & brand, not to exceed $25/mo. **Mail order** None	**Community and Mail Order** None

*Note: AWP = average wholesaler price; FUL = Federal Upper Limit; MAC= maximum allowable cost. Direct Supply Drug List: list of covered drugs consisting of certain maintenance drugs including specialty drugs, caloric supplements and substitutes and medical foods. Source: Centers for Medicare and Medicaid Services, 2006.

(Centers for Medicare & Medicaid Services 2006)

The CDT selection criteria used in the study were designed to restrict the sample to those CDTs that would be more appropriate for mail order. First, only CDTs in which the patient had utilized the drug for at least 90 days during the study period were selected. In practice, this is an ex-post criterion, as mail order pharmacists would not have this information prior to any decision to initiate mail order dispensing; this criterion is also consistent with mail order in that those medications discontinued immediately after the first refill were excluded. Second, the study utilized only those CDTs in which the patient maintained a minimum MPR of 0.5 during the study period. The study's CDT selection criteria allowed for the exclusion of drugs that were used during multiple periods of time (e.g. medication for allergy) and for short-term purposes (e.g. antibiotics). Third, the model assumes that the MPR associated with mail order pharmacy is the same as that observed in community pharmacy. In fact, however, mail order could increase the MPR given that patients have more medication available when 90-day supplies (vs. the 30-day supplies used in community pharmacy) are dispensed. Another assumption of the model is that mail order begins with the second fill; initiation of mail order at the first fill would further increase utilization given the high rate of therapy discontinuation that occurs before the second fill is dispensed. Fourth, the model also assumes that the drug supplies dispensed in mail order pharmacy are MPR-adjusted 90-day supplies. In fact, mail order programs can dispense supplies of 120 days or more. Increases in day supply would increase the difference in utilization in mail order vs. community pharmacy. And fifth, the baseline sample included all medications suitable for mail order, regardless of their classification and therapeutic category. To assess the validity of the criteria used to select these drugs, a sub-analysis was performed in relation to those drugs classified by the Ohio State Medicaid program as drugs for chronic use. A sub-analysis related to the dual-eligible population was also performed as this population of Ohio Medicaid patients are eligible for the new Medicare prescription drug program that began in 2006. A final sub-analysis included the subset of dual-eligible patients using drugs for chronic conditions. The three sub-analyses conducted in this study yielded similar increases in utilization derived from the use of mail order pharmacy.

Patients included in the study could have gaps in coverage; exclusion of these patients would misrepresent the potential effect of mail order pharmacy in the Medicaid program. Given a high turnover rate, periods of fluctuating coverage, and therapy discontinuation for enrollees associated with the Medicaid program, caution should be applied when generalizing these findings to other populations. In addition, the study only included drug reimbursement (drug product costs and dispensing fees); non-health care costs, such as patient costs to pick up medication at a local pharmacy, acquisition of medications through channels outside the scope of this study, and indirect costs were excluded from the analysis.

This study did not discount drug costs or adjust them for inflation. Discounting would have increased the cost differences resulting from mail order in that mail order payments are for three-month supplies as opposed to the one-month supplies used in community pharmacy. Adjusting drug costs for inflation would have reduced cost differences for the same reason. However, the study results would not change had these adjustments occurred.

Finally, this study does not evaluate the effect of increasing utilization on patients' outcomes. While higher utilization could result in higher patient compliance with drug treatments and increased access to drugs, this link has not been established in the available mail order pharmacy literature. Future research is needed to examine therapy discontinuation and estimation of drug wastage, resulting from medications that were not used because of tolerability issues, medication changes, ineffectiveness, and wastage attributed to other factors such as mail mishaps, spoilage, and patient failure to pickup rates, and an analysis of the impact of mail order pharmacy utilization on patient health outcomes. Future studies should also evaluate if the increase in utilization associated to mail order pharmacy could improve medication adherence and if adherence rates vary in different groups of Medicaid beneficiaries.

While the scope of this study is the Medicaid population, the results of the model related with increases in utilization can be also applicable to PBM commercial populations. Evaluation of mail order in a commercial population should evaluate if the reduction in costs per unit justifies the increase in utilization.

## Conclusion

The results of the simulation model used in this study indicate that mail order pharmacy increases drug utilization and can increase drug product cost if the cost per unit is not reduced accordingly. These results were achieved with CDT selection criteria that were designed to restrict the sample to only those CDTs for which the dispensing of 90-day supplies was appropriate.

Although increases in utilization may be beneficial for certain patients and may be linked to increases in medication adherence, policymakers should evaluate the utilization and cost effects that result from the implementation of mail order pharmacy programs. In addition, any program that increases the number of day supply dispensed to the patients should carefully target specific populations and therapies whose potential savings and/or health outcomes overcome the potential extra costs that result from increased utilization and wastage. Although, mail order is expected to have a large role in the Medicare Part D program, data are not yet available for analysis. Projections from this study reveal an increase in utilization attributed to mail order should be expected, and mail order services would be more appropriate for patients using maintenance drug therapies for chronic conditions for long periods of time (more than 2 years). In addition, mail order pharmacy services could facilitate access to medications for underserved areas and for elderly and disabled populations. This potential impact on access should be considered when evaluating the overall effect of mail order pharmacy.

## Competing interests

This study was funded by the Ohio Department of Job and Family Services and the Ohio Commission to Reform Medicaid as part of a larger analysis of the Ohio Medicaid pharmacy benefit. The authors developed the study's methodology, analyzed the data, and wrote this manuscript independently of the Ohio Medicaid program.

The author(s) declare that they have no competing interests.

## Authors' contributions

All authors actively participated in every step of the research process and in the development of this manuscript.

## Pre-publication history

The pre-publication history for this paper can be accessed here:


